# Divergent Picobirnaviruses in Human Feces

**DOI:** 10.1128/genomeA.00415-14

**Published:** 2014-05-15

**Authors:** Terry Fei Fan Ng, Everardo Vega, Nikola O. Kondov, Christopher Markey, Xutao Deng, Nicole Gregoricus, Jan Vinjé, Eric Delwart

**Affiliations:** a Blood Systems Research Institute, San Francisco, California, USA; b Department of Laboratory Medicine, University of California at San Francisco, San Francisco, California, USA; c National Calicivirus Laboratory, Centers for Disease Control and Prevention, Atlanta, Georgia, USA

## Abstract

The near-complete genomes of two picobirnaviruses (PBVs) in diarrheal stool samples, human picobirnaviruses D and E (HuPBV-D and -E), were genetically characterized. Their RNA-dependent RNA polymerase (RdRp) protein sequences had <66% identities to known PBVs. Due to a single nucleotide insertion, the open reading frame 2 (ORF2) in segment 1 of HuPBV-D was interrupted by a stop codon. A small stem-loop structure overlying the stop codon may result in translational readthrough into the rest of ORF2.

## GENOME ANNOUNCEMENT

Picobirnaviruses (PBVs) are small nonenveloped viruses with a genome consisting of two double-stranded RNA segments first identified in rats in 1988 (1) and partly sequenced first from rabbits in 1999 and then from humans in 2000 (2, 3). The first complete genome was described in 2005 from a human fecal sample (4). PBVs have been reported in fecal and respiratory samples in humans, other mammals, reptiles, and birds (5–9). The pathogenicity of PBVs remains uncertain. Currently, fewer than 15 segment 1 sequences have been reported and only one fully characterized human PBV genome sequence has been deposited in GenBank. Most PBV sequences in GenBank include only a short conserved region of the RNA-dependent RNA polymerase (RdRp) gene in segment 2 (6).

A total of 62 fecal samples from 15 outbreaks of unexplained diarrheal disease in humans with a typical viral gastroenteritis epidemiology (10) were analyzed by viral metagenomics. The samples had previously tested negative for established enteric viral pathogens (norovirus GI, GII, and GIV, sapovirus, astrovirus, rotavirus, adenovirus, and enterovirus). Unbiased deep sequencing using an Illumina Miseq platform was performed on enriched viral particles according to previously described protocols (11, 12). BLASTx was used to identify viral sequences based on translated protein sequence similarity to virus sequences in GenBank. Two divergent PBVs were identified in samples from two individuals from separate outbreaks, and their presence was confirmed by nucleic acid reextraction, reverse transcription-PCR (RT-PCR), and Sanger sequencing. Near-complete genomes from these two human picobirnavirus were assembled. Both viruses shared <66% identities with other PBVs in the RdRP proteins, including each other, reflecting highly distinct PBV species. To distinguish these sequences from the three existing human picobirnavirus segment 1 sequences (complete [GenBank accession no. AB186897] and partial [GU968923 and AF246941]), we named these human picobirnavirus D strain CDC23 (HuPBV-D-CDC23) and human picobirnavirus E strain CDC16 (HuPBV-E-CDC16).

For HuPBV-E, segment 1 was partially sequenced (2,056 bases), encoding a nearly complete capsid protein, whereas segment 2 of HuPBV-E was completely sequenced (1,717 bases). A near-complete genome was obtained for HuPBV-D, with 2,509 and 1,641 bases from segments 1 and 2, respectively, compared to 2,525 and 1,745 bases of the reference human PBV genome (NC_007026). Segment 1 normally contains 3 open reading frames (ORFs). Instead of the typical ORF2 of unknown function, the HuPBV-D ORF2 contained an extra base, resulting in an early stop codon in the middle of ORF2. We confirmed this insertion and premature ORF2 stop codon using both Illumina (30× coverage) and Sanger sequencing (4× coverage). A small stem-loop structure (5-bp stem and 9-nucleotide loop) was predicted to overlie the stop codon, indicating possible ribosomal frameshifting and translational readthrough.

With the use of RT-PCR with specific primers, only one patient from each diarrhea outbreak (among 2 and 3 tested individuals, respectively) tested positive for HuPBV-D and -E, suggesting a lack of direct association with diarrhea. A lack of association between PBV detection and diarrhea has been previously reported (3, 5), although given the wide genetic diversity of PBVs, it remains possible that certain genotypes are pathogenic in susceptible populations such as immunodeficient individuals (13).

### Nucleotide sequence accession numbers.

The genome sequences of HuPBV-D and -E have been submitted to GenBank under the accession numbers KJ663813 to KJ663816.

## References

[1] Pereira HG Flewett TH Candeias JA Barth OM 1988 A virus with a bisegmented double-stranded RNA genome in rat (*Oryzomys nigripes*) intestines. J. Gen. Virol. 69:2749–2754. 10.1099/0022-1317-69-11-2749 3053986

[2] Green J Gallimore CI Clewley JP Brown DW 1999 Genomic characterization of the large segment of a rabbit picobirnavirus and comparison with the atypical picobirnavirus of *Cryptosporidium parvum*. Arch. Virol. 144:2457–2465. 10.1007/s007050050658 10664398

[3] Rosen BI Fang ZY Glass RI Monroe SS 2000 Cloning of human picobirnavirus genomic segments and development of an RT-PCR detection assay. Virology 277:316–329. 10.1006/viro.2000.0594 11080479

[4] Wakuda M Pongsuwanna Y Taniguchi K 2005 Complete nucleotide sequences of two RNA segments of human picobirnavirus. J. Virol. Methods 126:165–169. 10.1016/j.jviromet.2005.02.010 15847933

[5] van Leeuwen M Williams MM Koraka P Simon JH Smits SL Osterhaus AD 2010 Human picobirnaviruses identified by molecular screening of diarrhea samples. J. Clin. Microbiol. 48:1787–1794. 10.1128/JCM.02452-09 20335418 PMC2863890

[6] Woo PC Lau SK Bai R Teng JL Lee P Martelli P Hui SW Yuen KY 2012 Complete genome sequence of a novel picobirnavirus, otarine picobirnavirus, discovered in California sea lions. J. Virol. 86:6377–6378. 10.1128/JVI.00686-12 22570247 PMC3372223

[7] Browning GF Chalmers RM Snodgrass DR Batt RM Hart CA Ormarod SE Leadon D Stoneham SJ Rossdale PD 1991 The prevalence of enteric pathogens in diarrhoeic thoroughbred foals in Britain and Ireland. Equine Vet. J. 23:405–409. 10.1111/j.2042-3306.1991.tb03751.x 1663866 PMC7185455

[8] Pereira HG Fialho AM Flewett TH Teixeira JM Andrade ZP 1988 Novel viruses in human faeces. Lancet **ii**:103–104 10.1016/s0140-6736(88)90032-3 2898672

[9] Masachessi G Martínez LC Giordano MO Barril PA Isa BM Ferreyra L Villareal D Carello M Asis C Nates SV 2007 Picobirnavirus (PBV) natural hosts in captivity and virus excretion pattern in infected animals. Arch. Virol. 152:989–998. 10.1007/s00705-006-0900-2 17245535

[10] Kaplan JE Gary GW Baron RC Singh N Schonberger LB Feldman R Greenberg HB 1982 Epidemiology of Norwalk gastroenteritis and the role of Norwalk virus in outbreaks of acute nonbacterial gastroenteritis. Ann. Intern. Med. 96:756–761. 10.7326/0003-4819-96-6-756 6283977

[11] Ng TF Kondov NO Hayashimoto N Uchida R Cha Y Beyer AI Wong W Pesavento PA Suemizu H Muench MO Delwart E 2013 Identification of an astrovirus commonly infecting laboratory mice in the US and Japan. PLoS One 8:e66937. 10.1371/journal.pone.0066937 23825590 PMC3692532

[12] Ng TF Marine R Wang C Simmonds P Kapusinszky B Bodhidatta L Oderinde BS Wommack KE Delwart E 2012 High variety of known and new RNA and DNA viruses of diverse origins in untreated sewage. J. Virol. 86:12161–12175. 10.1128/JVI.00869-12 22933275 PMC3486453

[13] Grohmann GS Glass RI Pereira HG Monroe SS Hightower AW Weber R Bryan RT 1993 Enteric viruses and diarrhea in HIV-infected patients. Enteric Opportunistic Infections Working Group. N. Engl. J. Med. 329:14–20 8099429 10.1056/NEJM199307013290103

